# Protein redox chemistry: post-translational cysteine modifications that regulate signal transduction and drug pharmacology

**DOI:** 10.3389/fphar.2014.00224

**Published:** 2014-10-06

**Authors:** Revati Wani, Asako Nagata, Brion W. Murray

**Affiliations:** ^1^Oncology Research Unit, Pfizer Worldwide Research and DevelopmentSan Diego, CA, USA; ^2^Worldwide Medicinal Chemistry, Pfizer Worldwide Research and DevelopmentSan Diego, CA, USA

**Keywords:** ROS, redox, cysteine oxidation, kinase, EGFR, subcellular trafficking, covalent drugs

## Abstract

The perception of reactive oxygen species has evolved over the past decade from agents of cellular damage to secondary messengers which modify signaling proteins in physiology and the disease state (e.g., cancer). New protein targets of specific oxidation are rapidly being identified. One emerging class of redox modification occurs to the thiol side chain of cysteine residues which can produce multiple chemically distinct alterations to the protein (e.g., sulfenic/sulfinic/sulfonic acid, disulfides). These post-translational modifications (PTM) are shown to affect the protein structure and function. Because redox-sensitive proteins can traffic between subcellular compartments that have different redox environments, cysteine oxidation enables a spatio-temporal control to signaling. Understanding ramifications of these oxidative modifications to the functions of signaling proteins is crucial for understanding cellular regulation as well as for informed-drug discovery process. The effects of EGFR oxidation of Cys_797_ on inhibitor pharmacology are presented to illustrate the principle. Taken together, cysteine redox PTM can impact both cell biology and drug pharmacology.

## INTRODUCTION

Reactive oxygen species (ROS) are the product of tightly controlled metabolic and cellular processes including mitochondrial respiration, cytokine and mitogen stimulations. They are also produced in response to external stressors such as pathogens, chemicals, and radiation (Bae et al., 1997; Dickinson and Chang, 2011; Bindoli and Rigobello, 2013). The ROS family broadly encompasses free radical (e.g., superoxide, ⋅O_2_^-^; hydroxyl, ⋅OH) and non-radical (e.g., peroxides) species (Cross and Templeton, 2006; Jacob et al., 2011). Superoxides and hydrogen peroxide are kinetically favorable for modifying select cysteine residues due to their regulated production (enzymatic, non-enzymatic), different rates of formation, half-lives and inherent reactivities relative to the more “toxic” hydroxyl radical (D’Autreaux and Toledano, 2007; Winterbourn, 2008; Drose and Brandt, 2012; Marinho et al., 2014). The key determinants of whether ROS function as secondary messengers for signal transduction or agents of cellular damage reside in the amount, type and cellular location of reactive species produced in response to its molecular trigger. Commonly understood aspects of redox modifications from ROS exposure include changes to the structure and conformation of proteins as well as inactivation of catalytic residues. However, the ability of specific oxidation reactions with ROS to alter interactions with small molecule inhibitors is poorly understood. The goal of this Perspective is to illuminate the complex interplay of localized production of ROS, protein oxidation events and drug interactions.

## THE CHEMISTRY OF CYSTEINE BIOLOGY: WHY CELLS PREFER CYSTEINE FOR OXIDATION?

The knowledge of sulfur chemistry is central to understanding how and why cysteine residues get preferentially oxidized during biological regulation. There are two sulfur-containing residues in the mammalian proteome, cysteine and methionine, of which cysteine constitutes 1–2% (Miseta and Csutora, 2000). The facile chemistry of cysteine stems from the electronic structure of its thiol group which enables multiple oxidation states (from -2 to +6) leading to an array of redox modifications (sulfenylation, SOH; sulfinylation, SO_2_H; sulfinylation, SO_3_H; glutathionylation, -SSG; protein disulfide formation; nitrosylation, etc.) with distinct chemical properties that contribute to signaling specificity (Wang et al., 2012). For instance, oxidation of the relatively hydrophobic cysteine residue to an oxoacid form (e.g., sulfinic acid) fundamentally changes it to a highly polar residue. Also, many types of cysteine oxidative modifications are readily reversible making them well-suited for initiating, amplifying, and terminating redox signaling relative to other residues (Bindoli et al., 2008). As such, this malleable chemistry confers unique capabilities to cysteine residues for sensing and transducing signaling by modifying the structure and function of proteins.

Oxidation of methionine, the other sulfur-containing amino acid, also leads to similar chemotypes as cysteine, however; the slower rate of methionine oxidation combined with rapid reduction by methionine sulfoxide reductase (MSR) minimizes its role in signaling regulation and suggests a role as redox scavengers (D’Autreaux and Toledano, 2007). Furthermore, reduced MSR expression in cells, aging individuals, and Alzheimer subjects is correlated with increased methionine oxidation which is consistent with an antioxidant function (Moskovitz, 2005; Schoneich, 2005; Moskovitz et al., 2011; Ringman et al., 2012). However, a recent study infers a role of methionine oxidation in signaling due to its proximity to Ser/Thr/Tyr residues which could impact phosphorylation networks (Rao et al., 2013). Further research on this intriguing correlation is necessary to establish the role of methionine oxidation as a signaling regulator. In addition to the sulfur-containing amino acids, oxidation of other amino acid residues can occur but they are not readily reversible making them ill-suited for initiating rapid on/off responses to upstream signals (Dickinson and Chang, 2011; Corcoran and Cotter, 2013). Cysteine oxidative modifications are therefore preferred for modulating signal transduction since they readily react with most ROS (Schoneich, 2011) to create a range of modifications that are largely reversible.

With an increased array of selective probes that label different redox chemotypes, our knowledge of proteins modified by oxidation is growing rapidly. Some of the known redox-modified kinases include MKK4 (Diao et al., 2010), PKC (protein kinase C) (Gopalakrishna and Jaken, 2000; Korichneva, 2005), Src (sarcoma kinase) (Giannoni et al., 2005), Akt2 (Wani et al., 2011), and more recently EGFR (epidermal growth factor receptor) (Paulsen et al., 2012; Schwartz et al., 2014). These modifications can be activating (PKC, Src, EGFR) or inhibitory (MKK4, AKT2). Redox-inactivation of phosphatases such as PTP1B, PTEN, and PP2A is also well-characterized (Kwon et al., 2004; Foley et al., 2007; Östman et al., 2011; Kitagishi and Matsuda, 2013). Other protein classes are also oxidatively modified including transcription factors (Surh et al., 2005; Cross and Templeton, 2006), ion channels; mitochondrial transporter proteins (O-Uchi et al., 2014), and cytoskeletal proteins (Landino et al., 2007; Farah et al., 2011). Therefore, selective oxidation of cysteine residues are post-translational modifications (PTM) with unique properties and capabilities.

## COMPARTMENTALIZED SIGNALING CONTRIBUTES TO SPECIFICITY OF REDOX REGULATION

Ligand stimulation of receptor tyrosine kinases (RTK) causes the translocation of the ligand – RTK complexes to clathrin-coated pits on the plasma membrane which bud off as endosomes and internalize (Carpenter and Liao, 2009; Murphy et al., 2009). From the endosome, RTK’s can traffick to lysosomes for degradation, to other intracellular destinations (e.g., cytoplasm, nucleus, mitochondria), or back to the membrane (recycling). EGFR endocytosis is a fairly well-understood process which also enables signaling from the endosome itself. (Paige Davis Volk and Moreland, 2014). Depending on the type of ligand (e.g., EGF, TGFα) and its dimerization partner, EGFR trafficking can either terminate in the lysosomes (following EGFR-ubiquitination) or at the membrane (recycling; Murphy et al., 2009; Sorkin and Goh, 2009). The mechanism of spatial regulation of EGFR signaling can be inferred from empirical studies that demonstrate how endoplasmic reticulum (ER)-based NOX4 produces ROS to locally inactivate the phosphatase PTP1B which allows the active receptor (phosphorylated EGFR) to recycle back to the membrane (Chen et al., 2008; Tomas et al., 2014). This diffusion-restricted process promotes spatial RTK signaling by (1) localizing ROS within the endosomal compartment and (2) preventing plasma membrane and cytoplasmic regulators (e.g., glutathione, antioxidant enzymes, trace metals) from interacting with the endosomal ROS. As such, the endosomal environment alters the local half-life of select ROS species to impart signaling selectivity.

Different oxidative modifications of the same protein, but at distinct subcellular locations, can add another layer of complexity to redox regulation. For example, recent biochemical studies on the reactivation of peroxide-inactivated PTP1B find different rates of recovery of its activity based on the reduction mechanism – enzymatic (thioredoxin, Trx) or by a non-protein thiol (glutathione, GSH). This study indicated that Trx selectively reduces lower order redox forms (sulfenic acid and sulfenyl amide) of PTP1B compared to GSH, which could also reduce some higher oxidized forms or sulfinyl derivatives of PTP1B. Interestingly, under comparable conditions Trx restored catalytic activity of PTP1B more efficiently than GSH (Parsons and Gates, 2013). If we extend this concept to cellular settings and assume that a fraction of PTP1B is localized within an endosome, then the stabilization of its sulfenyl form relative to the sulfinyl fraction would be largely determined by the dominant reducing agent in an endosome (Trx vs. GSH). Integrating this process with EGFR endocytosis suggests that the recycling rate of PTP1B would directly regulate the lifetime of active EGFR and therefore the amplitude of endosomal EGFR signaling. Also, the differential stability of individual sulfenylated/sulfinylated/disulfide-linked fractions of the EGFR molecules within endosomes can contribute to its signaling regulation.

Cysteine reactivity can be context-specific because the acid dissociation constant (p*K*_a_) of a cysteine residue’s thiol varies widely in proteins (p*K*_a_ 3–9) depending on many factors (Cremers and Jakob, 2013). As redox-sensitive proteins travel between different cellular compartments they are subjected to rapid changes in local pH, redox environment (vicinal protein/non-protein thiols, proximity to metal centers and positively charged residues) and solvent access that collectively affect the p*K*_a_ of their critical cysteine(s) (Roos et al., 2013). Thus, the local microenvironment affects protein thiol nucleophilicity which promotes spatially distinct, redox-mediated signaling. In addition, the different intrinsic rates of formation, diffusibility and stability of ROS (Bindoli et al., 2008) in these subcellular compartments adds a temporal control to redox regulation.

Post-translational modifications other than oxidation (e.g., phosphorylation, glycosylation, ubiquitination) can combine with redox regulation to impact cellular functions. For example, recent studies indicate that sodium arsenite-mediated oxidative stress promotes nuclear expulsion of the transcription factor Nurr1; a protein involved in survival of dopaminergic neurons with possible implications in neurodegenerative disorders such as Parkinson (García-Yagüe et al., 2013). As Erk1/2 phosphorylates Nurr1 (Lu et al., 2012), there is a possibility for combined phosphorylation and redox regulation in the nuclear/cytoplasmic trafficking of Nurr1. In case of EGFR, hydrogen peroxide treatment in cells is known to induce both EGFR phosphorylation and oxidation at Cys_797_ (sulfenylation; Paulsen et al., 2012) which independently enhance its catalytic activity. Hydrogen peroxide treatment of cells also enables trafficking of active EGFR to the perinuclear region by selective endocytosis (caveolar) and blocks ubiquitin-mediated degradation resulting in prolonged EGFR signaling (Khan et al., 2006; Sorkin and Goh, 2009). The narrow time window between peroxide-driven oxidation and phosphorylation could play a role in regulating membrane-based versus endosomal EGFR signaling. Therefore, resolving the time courses of individual PTMs can be crucial to understanding the functional responses of signaling proteins. Taken together, dynamic variations in the redox environment, nucleophilicity of the cysteine thiol, and the interplay with other PTMs during trafficking collectively determines the stability of a select protein oxidation event in a subcellular compartment thus resolving the kinetics of spatio-temporal signaling.

## DOES OXIDATION MODULATE PROTEIN CONFORMATION?

The structure and conformation of proteins are central to their biological function. Redox regulation of protein conformation can be achieved by multiple mechanisms (1) cysteine oxidation leading to disulfide bond formation, (2) cysteine-dependent metal cofactor interactions, and (3) cysteine modifications that alter the topography of a protein (e.g., sulfinic acid, sulfenamide). Disulfide bond formation is a well-established mechanism stabilizing higher order protein structures occurring in the oxidizing environment of ER during protein maturation (Zhong and Wright, 2013). These structural disulfide bonds are characterized by very low redox potentials (as low as -470 mV; Fan et al., 2009; Wouters et al., 2010) and will not be addressed in this Perspective. Another category of disulfide bond has emerged more recently, redox-active disulfide bonds, which are energetically distinct from structural disulfides (redox potential from -95 to -330 mV) and are used in dynamic regulatory mechanisms (Fan et al., 2009; Wouters et al., 2010). For example, integrins require reduced cysteine residues to achieve an active conformation and are inactivated upon disulfide bond formation (Yan and Smith, 2000, 2001; Chigaev et al., 2004). DTT-induced reduction (*in vitro*) of the candidate disulfide bonds (Cys_406_/Cys_655_, Cys_457_/Cys_495_) and disulfide rearrangements of other cysteine residues drives the integrin conformation to its active state. This mechanism of integrin activation has been targeted by a small molecule agent to re-sensitize drug-resistant acute myelogenous leukemia cells to chemotherapy (Layani-Bazar et al., 2014). Another biological relevant catagory of disulfide bond is mixed disulfides (e.g., glutathionylation). Actin glutathionylation studies illustrate the functional significance of this type of modification. Ischemia-reperfusion induces glutathionylation of actin at Cys_374_ which blocks polymerization of α-actin filaments, decreases actomyosin ATPase activity (Pizarro and Ogut, 2009), and reduces muscle contractility. An array of sarcomeric proteins are additionally shown to be oxidized, (e.g., troponin, tropomyosin) suggesting that cardiac contractility is regulated by a network of redox-modified proteins (Steinberg, 2013).

The well-studied mechanism of redox-induced conformational change involves metal-derived redox reactions that occur frequently with changes in cellular thiol balance (Ilbert et al., 2006; Fan et al., 2009). Conformational changes resulting from Zn release in proteins such as Hsp33 (Ilbert et al., 2006), metallothionein (Oteiza, 2012), thioredoxin 2 from *Escherichia coli* (El Hajjaji et al., 2009), PKC (Zhao et al., 2011), and antisigma factor RsrA (Heo et al., 2013) routinely occur following oxidative stress. The coordinating cysteine residues in the zinc finger cluster form intramolecular disulfides releasing Zn which leads to unfolding of the redox-responsive region in Hsp33. This exposes both the dimerization interface and substrate binding site to enhance folding of the target proteins and alternatively prevents their aggregation until the cellular stress (e.g., redox, temperature) is relieved (Ilbert et al., 2006). Kinetic studies measuring reactivities of zinc finger peptides with peroxide and oxygen suggest that binding of Zn to the reduced Hsp33 monomers minimizes the nucleophilicity or thiolate form (S-) of the coordinating cysteine residues and curbs redox reactions (Bourles et al., 2011). Another example is the zinc metalloprotein metallothionein which is redox-regulated to affect zinc transfer and homeostasis (Oteiza, 2012).

Specific redox modifications of cysteine residues can change their chemical properties, alter protein topography, and contribute to biological regulation. For example, oxidation of PTP-1B catalytic cysteine (Cys_215_) to sulfenamide via a sulfenic acid intermediate induces major structural rearrangements of two loops in its active site and is shown to be essential for substrate recognition and catalysis (Brandes et al., 2009). Taken together, redox-regulated conformational changes in signaling proteins occur by an array of mechanisms and play important roles in biological regulation.

## ONCOLOGY TARGETS OF REDOX: KINASES WITH A HINGE REGION CYSTEINE IN THEIR CATALYTIC DOMAIN

**FIGURE 1:**
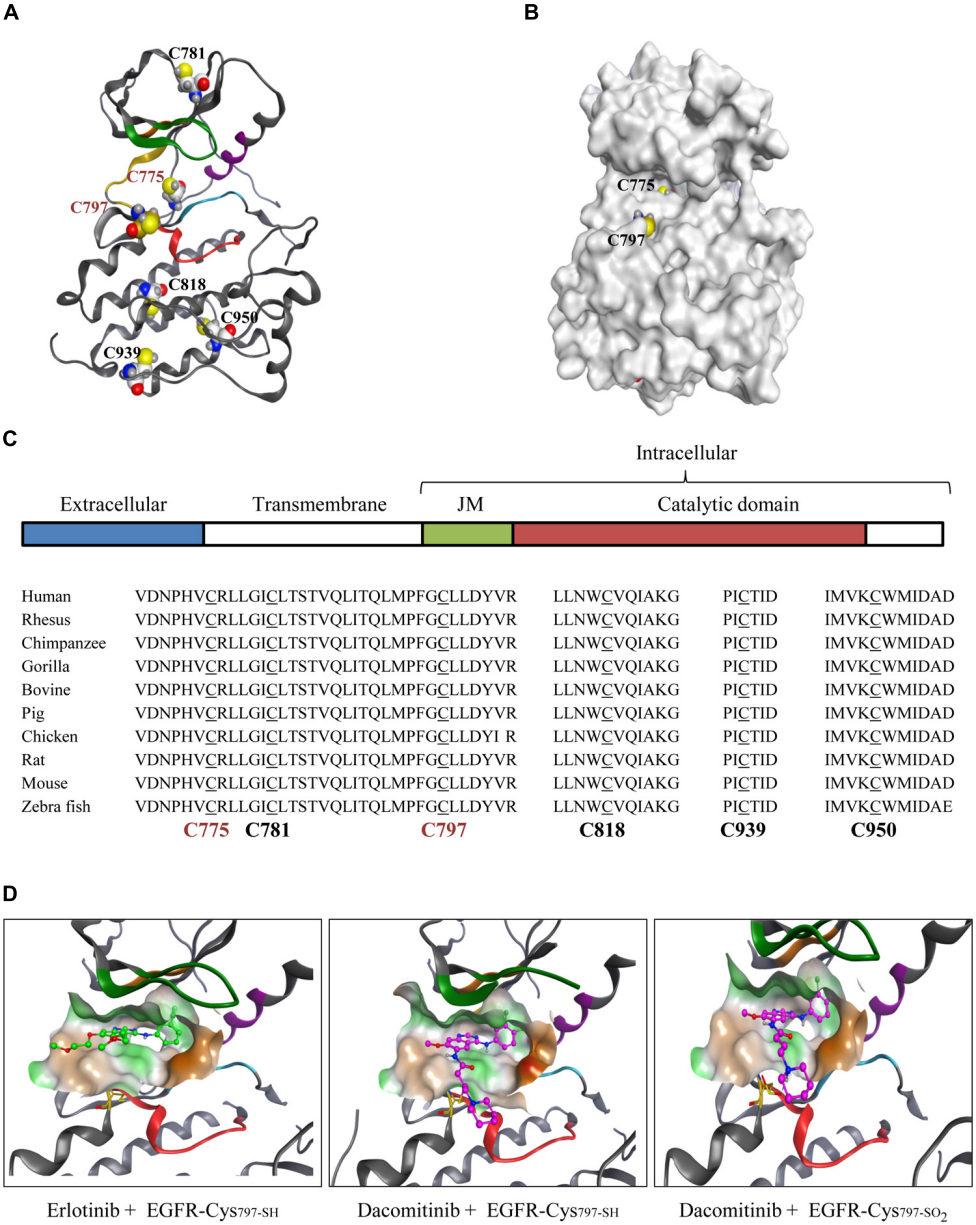
**Pharmacological role of evolutionarily conserved EGFR-Cys_**797**_. (A)** Catalytic domain of WT EGFR depicting all six cysteine residues. **(B)** Surface structure of WT EGFR shows Cys_775_ and Cys_797_ as the two solvent-accessible cysteine residues (PDB 1M14). **(C)** Schematic representation of EGFR domains: ligand binding extracellular domain (blue), the transmembrane domain (green), juxtamembrane region, JM (white) and the catalytic domain (red). Phylogenetic analysis of catalytic domain cysteine residues for the indicated species using the Uniprot Clustal O sequence alignment tool. Solvent-exposed cysteine residues are in red font. **(D)** Binding of small molecule inhibitors to the WT EGFR active site is shown in context of Cys_797_ (yellow; PDB 1M17). Reversible inhibitor erlotinib (green) binding to WT EGFR **(left)**, covalent inhibitor dacomitinib (purple) binding to the reduced thiol Cys_797-SH_ of WT EGFR **(middle)** and the sulfinylated Cys_797-SO2_ WT EGFR **(right)**.

One of the best known non-catalytic cysteine nucleophiles occurs in the catalytic domain of a group of 11 protein kinases that includes EGFR. A brief overview of the known pathophysiological roles of these kinases along with a list of investigational and approved drugs from the most recent literature is included (**Table 1**). In physiological conditions, EGF ligand stimulation induces receptor dimerization followed by autophosphorylation of activation loop tyrosine residues, recruitment of the adaptor proteins, and activation of downstream signaling pathways to regulate fundamental cellular processes. In the disease setting, EGFR can be up-regulated, mutated, and constitutively activated in a range of solid tumors and leukemias. (Seshacharyulu et al., 2012; Sasaki et al., 2013; Arteaga and Engelman, 2014). Phylogenetic analysis of the six cysteine residues distributed throughout the EGFR catalytic domain demonstrates a strong evolutionary conservation (). Although zebrafish share only about 40% sequence identity with human EGFR, the catalytic domain cysteines remain conserved which suggests a strong biological relevance of these residues. Of the six residues, four (Cys_781_, Cys_818_, Cys_939_, and Cys_950_) possess limited solvent access. Two of these four cysteines are deeply buried in the hydrophobic pocket while the other two are at the surface but their side chains point inward thus restricting solvent access. The remaining two residues (Cys_797_, Cys_775_) are in close proximity to solvent channels (**Figure 1B**) and are likely to undergo redox modifications. Historically, one of these solvent-exposed cysteines has been targeted for therapeutic intervention (Cys_797_).

**Table 1:** Protein kinases with a nucleophilic cysteine residue in the hinge region of the catalytic domain.

Kinase	Biological function	Disease relevance	Approved and investigational drugs
EGFR	Proliferation, development and differentiation of squamous epithelial cells, regulates cell cycle, cytoskeletal reorganization, and cell migration	NSCLC, colorectal cancer, breast cancer, cervical cancer, prostate cancer, renal cell carcinoma, bladder cancer, head, and neck cancer	Dacomitinib; Afatinib; Iressa (Gefitinib); Tarceva (Erlotinib); Erbitux (Cetuximab); Tykerb (Lapatinib); Vectibix (Panitumumab); Caprelsa (Vandetanib); AZD9291; CO-1686
HER2/ErbB2	Regulates proliferation, differentiation, and cytoskeletal rearrangement in cells, regulates transcription and cell cycle, and cell migration	Breast cancer, glioma, ovarian cancer, lung cancer, hereditary diffuse gastric cancer	Dacomitinib; Afatinib; Herceptin (Trastuzumab); Kadcyla (ado-trastuzumab emtansine); Perjeta (Pertuzumab); Tykerb (Lapatinib)
HER4/ErbB4	Proliferation, development, and differentiation of cardiomyocytes; migration of neural crest cells and mammary epithelial cells	Schizophrenia, breast cancer (tumor suppressor), medulloblastoma, viral leukemia	Dacomitinib; Tykerb (Lapatinib)
BTK	Development, proliferation, and activation of myeloid cells, mast cells and B-cells; regulation of apoptotic signaling; transcriptional regulation of NF-kB	X-linked agammaglobulinemia, multiple myeloma, non-hodgkin lymphoma, immunodeficiency, wiskott-aldrich syndrome,	Ibrutinib (PCI-32765) GDC-0834; CGI-560; HM-71224; CC-292; ONO-4059; CNX-774; LFM-A13
ITK	Regulates T-cell signaling, trafficking, proliferation, and viral replication	Inflammatory skin diseases such as atopic dermatitis, psoriasis, allergic contact dermatitis	Ibrutinib (PCI-32765)
BMX	Cell differentiation, inflammatory signaling, motility, survival, and angiogenesis	Hepatocellular carcinoma, prostatic intraepithelial neoplasia, ischemia, leukemia, lung cancer, arthritis	Ibrutinib (PCI-32765); MK2206; Canertinib (CI-1033)
BLK	Regulates B cell development, differentiation and activation; modulates beta cell function in pancreatic islets	Maturity-onset diabetes of the young 11 (MODY-11), motor neuropathy, scleroderma, arthritis, lupus, acute lymphoblastic leukemia	None to date
TXK	Growth, development, differentiation and activation of T lymphocytes; actin reorganization; regulates cytokine production in TH1 cells	Rheumatoid arthritis, Behcet’s disease, bronchial asthma, atopic dermatitis	Ibrutinib (PCI-32765)
TEC	T cell development, differentiation and cytokine production (IL2); regulates growth and differentiation of myeloid cells	X-linked agammaglobulinemia, autoimmune conditions, inflammatory diseases, arthritis, congenital fibrosarcoma, hepatitis, squamous cell carcinoma, lymphedema	Ibrutinib (PCI-32765)
JAK3	Cell growth, development and differentiation; regulates hematopoiesis and gene expression via STAT signaling	Psoriasis, rheumatoid arthritis, severe combined immunodeficiency (autosomal recessive for T^-^ cell –ve/B cell +ve/NK cell –ve)	Tofacitinib, VX-509
MKK7	Activated in response to cellular stresses, proinflammatory cytokines and regulates apoptotic signaling in neurons. Activates SAPK/JNK pathways in cells, regulates toll-like receptor signaling pathways, regulates proliferation of hematopoietic cells, regulates differentiation of helper T cells (Th) to Th1	Advanced stage prostatic tumors, schizophrenia, hypertrophic cardiac diseases (animal models)	None to date

*Redox modifications have been reported for a subset of these proteins (e.g., EGFR, BTK, JAK3).*

Covalent kinase inhibition is a well-known pharmacological strategy that targets non-catalytic nucleophilic residues (e.g., Cys_797_ of EGFR) with electrophilic inhibitors. This approach was pursued to create highly potent drugs with prolonged pharmacological effects and enhanced selectivity (Singh et al., 1997; Schwartz et al., 2014). The most prevalent approach uses a Michael addition reaction of a nucleophilic cysteine thiol with the electron-deficient β-carbon of the Michael acceptor (MA) substituent of a covalent inhibitor. Several FDA-approved covalent drugs utilize this approach (e.g., afatinib, ibrutinib; **Table 1**). EGFR-Cys_797_ is capable of undergoing multiple redox modifications (sulfinylation and glutathionylation) that can impact the potency of targeted drugs (Schwartz et al., 2014). Our studies show that reversible binding affinity of EGFR inhibitors is highly variable dependent on the inhibitor structure, the type of redox modification of Cys_797_, and the protein context (type of mutation). For example, biochemical affinity of the covalent inhibitor dacomitinib decreased by about 30-fold when the L858R EGFR mutant was glutathionylated (*K*_i_ from 0.7 to 21 nM) but was unaffected by sulfinylation (*K*_i_ from 0.7 to 1.0 nM). A similar trend was observed with the drug-resistant L858R/T790M EGFR mutant, however, the loss in affinity was more substantial for the glutathionylated protein (>50-fold). The active sites of WT and L858R mutant EGFR are structurally very similar, hence biochemical affinities of their covalent drugs are expected to be comparable. Modeling studies of dacomitinib interactions with WT EGFR and WT EGFR sulfinylated at Cys_797_ do not predict significant loss of interactions between the inhibitor MA and sulfinylated EGFR-Cys_797_ which is consistent with the modest decrease in affinities (**Figure 1D**). However, the more substantial loss of dacomitinib interactions with glutathionylated EGFR-Cys_797_ may occur due to reduced access of the inhibitor to the ATP-binding pocket therefore interfering with both covalent and non-covalent interactions. For non-covalent drugs such as erlotinib that do not require EGFR-Cys_797_ interactions, the loss of affinity was modest for the both EGFR mutants (about twofold). As such, redox modifications of protein kinases can result in a wide range of pharmacological effects and be inhibitor-specific.

In cellular environments, the pharmacology of covalent inhibitors can be more complex due to spatially distinct and temporally controlled redox signaling mechanisms. The rate of formation and stability of oxidized-EGFR can vary depending on the local redox environment within subcellular compartments. Therefore, an inhibitor may have different interactions (i.e., potency) with a given protein based on its subcellular location. *As such, two EGFR inhibitors may have a different spectrum of pharmacological effects due to their individual abilities to bind to different redox forms of EGFR.* This new frontier of drug pharmacology should be exploited to create more effective and better-tolerated drugs.

## SUMMARY

Despite their low abundance in the mammalian proteome, cysteine residues have emerged as important effectors of signaling regulation in both healthy cells and pathophysiological states. The chemistry of cysteine residues enables many types of post-translational oxidation reactions in cells with unique roles relative to the other amino acids. The conformational changes induced by redox modifications in signaling proteins can regulate biological outcomes. The spatial networks created by compartmentalized trafficking of redox-active proteins between distinct redox microenvironments impart selectivity to the kinase signaling. Temporal separation of the localized redox networks further refines signaling regulation. Specific cysteine oxidation events can also modulate small molecule inhibitor interactions and impact the clinical performance of these drugs. As such, redox analysis is necessary to gain a deeper understanding of biology and to design more effective drugs.

## AUTHOR CONTRIBUTIONS

Both authors contributed to this work and approve the version to be published and are also accountable for all aspects of the work.

## Conflict of Interest Statement

The authors declare that the research was conducted in the absence of any commercial or financial relationships that could be construed as a potential conflict of interest.
